# The Algal Polysaccharide Ulvan and Carotenoid Astaxanthin Both Positively Modulate Gut Microbiota in Mice

**DOI:** 10.3390/foods11040565

**Published:** 2022-02-16

**Authors:** Kunal Pratap, Marwan E. Majzoub, Aya C. Taki, Socorro Miranda Hernandez, Marie Magnusson, Christopher R. K. Glasson, Rocky de Nys, Torsten Thomas, Andreas L. Lopata, Sandip D. Kamath

**Affiliations:** 1 Molecular Allergy Research Laboratory, Discipline of Molecular and Cell Biology, College of Public Health, Medical and Veterinary Sciences, James Cook University, Townsville, QLD 4811, Australia; kunal.pratap@my.jcu.edu.au (K.P.); andreas.lopata@jcu.edu.au (A.L.L.); 2 Australian Institute of Tropical Health and Medicine, James Cook University, Townsville, QLD 4811, Australia; socorro.mirandahernandez1@jcu.edu.au; 3 Center for Molecular Therapeutics, James Cook University, Townsville, QLD 4811, Australia; 4 Centre for Marine Science and Innovation, School of Biological, Earth and Environmental Sciences, Faculty of Science, The University of New South Wales, Sydney, NSW 2052, Australia; m.majzoub@unsw.edu.au (M.E.M.); t.thomas@unsw.edu.au (T.T.); 5 Department of Veterinary Biosciences, Melbourne Veterinary School, Faculty of Veterinary and Agricultural Sciences, The University of Melbourne, Parkville, VIC 3010, Australia; aya.taki@unimelb.edu.au; 6 Te Aka Mātuatua—School of Science, Environmental Research Institute, University of Waikato, Tauranga 3110, New Zealand; marie.magnusson@waikato.ac.nz (M.M.); christopher.glasson@waikato.ac.nz (C.R.K.G.); 7 School of Marine Science and Tropical Biology, James Cook University, Townsville, QLD 4811, Australia; rocky.denys@jcu.edu.au; 8 Tropical Futures Institute, James Cook University Singapore, Singapore 387380, Singapore

**Keywords:** algae, polysaccharide, carotenoid, ulvan, astaxanthin, mouse model, microbiota

## Abstract

The intestinal microbial community (microbiota) is dynamic and variable amongst individuals and plays an essential part in gut health and homeostasis. Dietary components can modulate the structure of the gut microbiota. In recent years, substantial efforts have been made to find novel dietary components with positive effects on the gut microbial community structure. Natural algal polysaccharides and carotenoids have been reported to possess various functions of biological relevance and their impact on the gut microbiota is currently a topic of interest. This study, therefore, reports the effect of the sulfated polysaccharide ulvan and the carotenoid astaxanthin extracted and purified from the aquacultured marine green macroalgae *Ulva ohnoi* and freshwater green microalgae *Haematococcus pluvialis,* respectively, on the temporal development of the murine gut microbiota. Significant changes with the increase in the bacterial classes *Bacteroidia*, *Bacilli*, *Clostridia*, and *Verrucomicrobia* were observed after feeding the mice with ulvan and astaxanthin. Duration of the treatments had a more substantial effect on the bacterial community structure than the type of treatment. Our findings highlight the potential of ulvan and astaxanthin to mediate aspects of host-microbe symbiosis in the gut, and if incorporated into the diet, these could assist positively in improving disease conditions associated with gut health.

## 1. Introduction

The gut microbiota plays an important role in human health and well-being. An imbalance or dysbiosis of the gut microbiota has been associated with several chronic and inflammatory non-communicable diseases, such as obesity, type-2 diabetes, and inflammatory bowel disease [1]. Diet and dietary fibers play a central role in maintaining gut homeostasis, as bacterial populations use them to produce short-chain fatty acids (SCFAs) and other molecules, which interact with the intestinal mucosal barrier and assist in immune tolerance [2,3]. Different dietary supplements involving a range of macromolecules, such as polysaccharides, are efficacious in promoting the growth of beneficial bacteria to produce immune-boosting metabolites [2,3,4]. Dietary polysaccharides are mostly found in plant-based food products; however, untapped resources, such as both marine and freshwater algae, are increasingly gaining interest as a source of polysaccharides [5,6,7]. Algal polysaccharides from different origins are known for their immune-modulating properties and suppressing inflammatory responses [2,8].

Properties of a polysaccharide, such as glycosidic linkages, molecular weight, monosaccharide composition, and sulfate content vary between polysaccharides and algal species [8,9]. However, unlike terrestrial plant polysaccharides, many algal polysaccharides are sulfated (e.g., fucoidan from brown, carrageenan, and agar from red seaweed), contributing to their various structural properties and biological functions [10]. For example, *Ulva ohnoi* is a marine green macroalga rich in sulfated ulvan, composed of sugars (i.e., rhamnose and xylose) and other components, such as different uronic acids [11,12]. As such, macro- and microalgal-derived polysaccharides have been explored for different purposes, such as alternative food products and nutraceuticals, or for their anti-inflammatory [10,13], antioxidant [13,14], and immunomodulatory activity [15,16].

Microalgae contain an abundance of various pigmented components, such as carotenoid xanthophylls and chlorophylls [17], that have also been reported to have beneficial bioactivities in health applications [18]. Astaxanthin (3,3′-dihydroxy-ß-carotene-4,4′-dione) is a carotenoid extracted from the freshwater microalga *Haematococcus pluvialis,* but is also found as a major xanthophyll component in other microalgae and yeast [16,19]. It is also present in seafood, including shrimp, lobster, and salmon, after acquiring it through feeding on microorganisms that produce astaxanthin [16,20]. Astaxanthin is a secondary carotenoid easily distinguishable by its bright red color and is structurally related to other carotenoids, such as β-carotene and lutein. Astaxanthin is a ketocarotenoid, meaning it contains hydroxyl and carbonyl functional groups, making it a prime target for exploring the antioxidant properties in biomedical applications [20].

Ulvan and astaxanthin have been described for their beneficial bioactive properties; however, reports on their impact on the microbiota are scarce [21,22], particularly for purified extracts. We expect that the incorporation of ulvan and astaxanthin into the diet could have an overall impact on the gut microbiota. Therefore, this study investigated the effect of feeding the sulfated polysaccharide ulvan from *U. ohnoi* and the carotenoid astaxanthin from *H. pluvialis* on the murine gut microbiota using 16S rRNA gene sequencing. BALB/c mice were fed with either a control diet, ulvan, or astaxanthin for 28 days. Ulvan and the astaxanthin treatment changed the bacterial community structure compared to the naïve group of mice, increasing the relative abundance of classes *Bacteroidia*, *Bacilli*, *Clostridia*, and *Verrucomicrobia*. The study outcomes help us understand the potential impact of polysaccharides and carotenoids on the mouse gut microbiota, which may play an essential role in maintaining gut homeostasis, and subsequently, their therapeutic potential in inflammatory gut diseases.

## 2. Methods

### 2.1. Animals

BALB/c, female, 6- to 8-week-old mice (total *n* = 15, *n* = 5 per group) were obtained from the Australian Institute of Tropical Health and Medicine (AITHM) at James Cook University, Townsville, Australia. Mice were maintained on a 12 h light/dark cycle in individually ventilated cages (Tecniplast, Lane Cove West, NSW, Australia). This study and all protocols were carried out following the recommendations from an independent ethics committee for animal experimentation (Ethics ID: A2524).

### 2.2. Procurement of Ulvan and Astaxanthin

*Ulva ohnoi* was grown at scale in-house in a land-based aquaculture system at James Cook University, as described previously [23]. The extraction of ulvan from *U. ohnoi* was performed by Marinova Pty Ltd. (Cambridge, TAS, Australia) using a proprietary mil aqueous process. Purification of the resulting extract was performed as described previously [10]. Astaxanthin from *H. pluvalis* was supplied by Pacific Biotechnologies Pty Ltd. (Abbotsford, VIC, Australia). Briefly, crude ulvan was dissolved in Type 1 water, vacuum-filtered (Filtech, 453), and then diafiltered with five volumes of Type 1 water using an Äkta flux 6 system equipped with a 10,000 NMWC filter, UFP-10-E-4 × 2MA. Protein was removed from the retentate using anion exchange chromatography (AEC) (Äkta Pure 150L equipped with a single wavelength UV-detector at 280 nm). The column (XK 50/30 column, GE Healthcare Life Sciences) was equilibrated as follows; Type-1 water, 5 column volumes (CV); 2 M NaCl, 5 CV; Type 1 water, 5 CV, and the retentate was eluted using a stepwise gradient (0 M NaCl, 2 CV; 0–0.5 M NaCl, 2 CV; 0.5–1 M, 2 CV; 1–1.75 M NaCl, 3 CV; 1.75–2 M NaCl, 5 CV) at a flow rate of 20 mL min^−1^). Fractions containing uronic acids (detected calorimetrically using the m-phenyl-phenol method with glucuronic acid as standard) were pooled and diafiltered to concentrate until the permeate conductivity was <5 µS cm^−1^ [10]. This purified ulvan was freeze-dried and then milled to a fine powder using mortar and pestle.

Astaxanthin from *H. pluvalis* was supplied by Pacific Biotechnologies Pty Ltd. (Abbotsford, VIC, Australia).

### 2.3. Feeding Regimen

**Figure 1:**
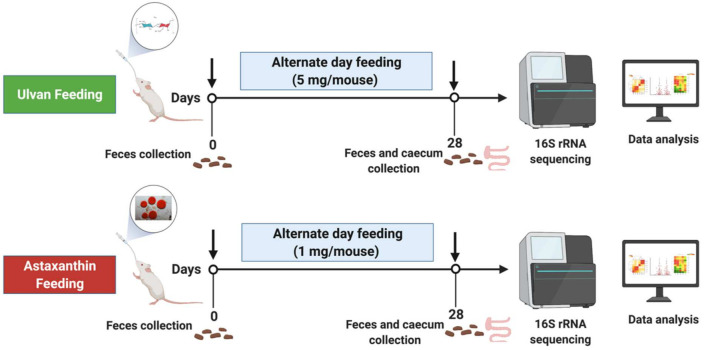
Feeding of ulvan and astaxanthin to BALB/c mice. Timeline depicting the feeding regimen of ulvan and astaxanthin on alternate days for 28 days. Feces pellets were collected on Day 0 and Day 28 and caecum samples on Day 28 and analyzed for the microbiome.

Six- to eight-week-old mice were randomly separated into three groups: naïve, ulvan, and astaxanthin. Five mice per group were distributed and housed together during the experiment. The groups received purified ulvan extract (5 mg/mouse) and astaxanthin doses (1 mg/mouse) respectively via intragastric gavage every second day for 28 days (Figure 1). Purified ulvan was prepared as 5 mg ulvan/200 μL PBS (25 μg ulvan/μL). The astaxanthin was procured as an emulsified solution in medium-chain triglyceride (MCT) oil. For our experiment, astaxanthin was prepared as 5 μL of emulsified astaxanthin solution in 195 μL of PBS with 1 mg astaxanthin (5 μg astaxanthin/μL) in solution. Drinking water and irradiated food pellets of soy-free rat and mouse reformulated diet (Specialty feeds, Australia) were fed to the mice *ad libitum*.

### 2.4. Sample Collection

Mice were monitored briefly after feeding for any physical discomfort. Fecal samples were collected in DNase and RNase-free tubes on Day 0 and Day 28, two hours after feeding the ulvan and astaxanthin. After 28 days, the mice were sacrificed using CO_2_ asphyxiation. Caecum samples with intact fecal matter were collected and snap-frozen in liquid nitrogen. All samples were stored at −80 °C (Figure 1).

### 2.5. Microbial Community Analysis

#### 2.5.1. DNA Extraction and 16S rRNA Gene Amplification and Sequencing

Total DNA from the fecal and caecum samples (weight 200 mg) of mice was extracted using the DNeasy Powersoil kit following the manufacturer’s instructions (Qiagen, Hilden, Germany). The bacterial community composition of the samples was investigated by sequencing the V3-V4 hypervariable region of the 16S rRNA gene using the universal primers 341F & 785R as previously described [24]. Briefly, the amplification was performed for a final reaction volume of 50 μL per sample containing 2X Master Mix (Econotaq^®^ PLUS GREEN, Astral Scientific, Sydney, Australia), 10 μM of each primer, 20 ng/µL of template DNA. The cycling conditions for PCR included initial denaturation at 94 °C for 2 min, followed by 35 cycles of denaturation at 94 °C for 30 s, annealing at 55 °C for 30 s, extension at 72 °C for 40 s, and a final extension at 72 °C for 7 min. The amplicons were quality-checked by a gel electrophoresis system and then paired-end sequenced (2 × 300 bp) on a MiSeq platform at the UNSW Ramaciotti Centre for Genomics, as described in the User Guide (Illumina 2013).

#### 2.5.2. Sequencing Data Analysis

The sequences of the V3-V4 region were analyzed as described by Wemheuer and Wemheuer (2017) [25]. Briefly, quality-filtering and trimming of the paired-end reads were done using TRIMMOMATIC version 0.36 [26]. USEARCH version 11.0.667 [27] was used to merge, read, and quality-filter them, excluding sequences with <250 or >550 nucleotides, in addition to sequences with more than one ambiguous base or an expected error of more than 1. Filtered sequences were denoised and clustered into amplicon sequence variants (ASV) using the USEARCH-UNOISE algorithm. The chimera detection was performed using UCHIME version 4.1 [28] with the SILVA SSURef 132 NR database [29]. The ASV obtained were taxonomically classified by BLASTN [30] against the SILVA database. The ASV table was filtered to remove all non-bacterial, non-BLAST aligned, and singleton ASVs.

#### 2.5.3. Community and Statistical Analysis

To assess the species richness in the samples, we generated rarefaction curves using the rarecurve function of the vegan package in R (R version 3.5.3, Vienna, Austria), as described previously [31]. For subsequent analysis, samples were normalized to 22,900 counts per sample. The alpha diversity in the sample population was calculated as a measure of observed species, ASV richness, and Shannon index in R using the rarefy function in the vegan package for community ecology analysis [32]. Briefly, alpha diversity is an indicator of diversity in a single sample measured using ASV richness. ASV richness is the number of ASVs with at least one read for each sample estimated using the Shannon index, that is, an estimate of the diversity of the species in each sample [33]. A two-way ANOVA test in GraphPad Prism 8.0.2 (San Diego, CA, USA) followed by Tukey’s pairwise comparisons test was used to determine the significance between the different groups; a *p*-value < 0.05 was considered significant.

ASV tables were imported into PRIMER (primer-e, Albany, Auckland, version 6) [34] for multivariate analysis of microbial communities to compare the community structure (i.e., relative abundance data). Bray–Curtis similarity coefficients were calculated using square-root transformed ASV abundances, and the resulting similarity matrix was visualized using non-metric, multidimensional scaling (nMDS). Permutational multivariate analysis of variance (PERMANOVA) [35] with 9999 random mutations was used to test the effect of sample type, treatment, and time on microbial communities in mouse fecal samples. ‘Sample type’ (“fecal” or “caecum”) was a fixed factor, ‘Treatment’ (“naïve”, “astaxanthin” and “ulvan”) was a fixed factor, and ‘Time’ (“Day 0” and “28”) was a fixed factor.

## 3. Results

### 3.1. Bacterial Community Recovery from Samples

We used a 16S rRNA gene-based analysis to assess bacterial communities from mouse fecal and caecum samples. After quality filtering, there were a total of 1,625,935 sequencing reads clustered into 341 ASVs. Rarefaction analysis and an average Good’s coverage of 99.95% ± 0.04% indicated that the given sequencing effort recovered the majority of the bacterial diversity in the samples (Figure S1).

### 3.2. Diversity and Richness of Microbiota in Ulvan and Astaxanthin Fed Groups

**Figure 2:**
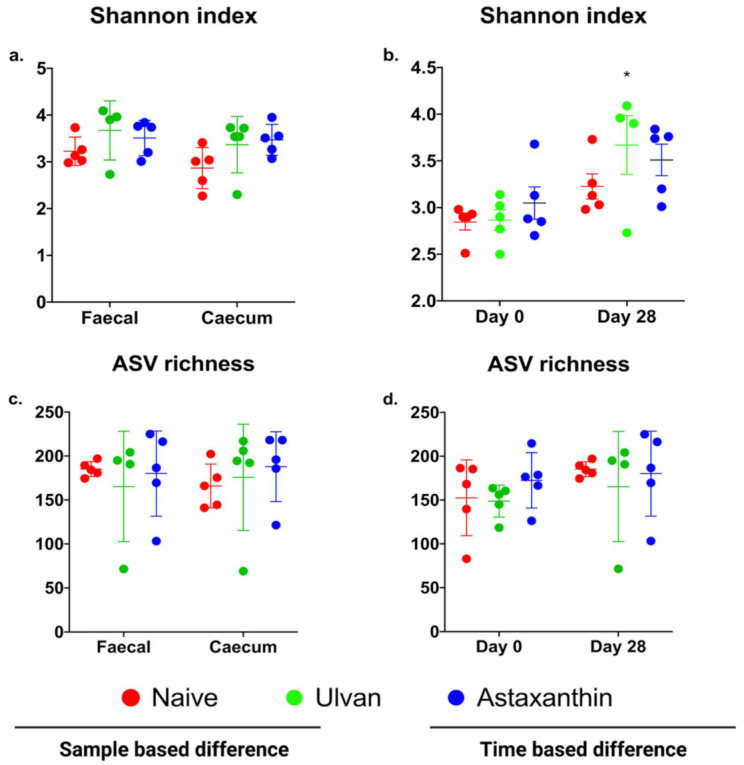
Differences in the diversity and richness between the fecal and caecum samples based on the type of sample and time (Day 0 and 28), as shown using the Shannon index and ASV richness. Shannon diversity index (**a**,**b**) and ASV richness (**c**,**d**) data based on sample and time difference are presented as mean ± SEM.

There was no statistical support for differences in diversity or richness between the fecal and caecum samples on day 28 (*p* > 0.05) (Figure 2a,c). There was an increase in diversity for day 28 samples compared to day 0, which was more pronounced for the ‘ulvan’ treatment (*p* = 0.0114) (Figure 2b). There was no statistical support for differences in richness between the treatments on days 0 and 28 (*p* > 0.05) (Figure 2d).

### 3.3. Algal Polysaccharide Feeding Affect Bacterial Community Structure and Relative Abundance of Bacterial Diversity

**Figure 3:**
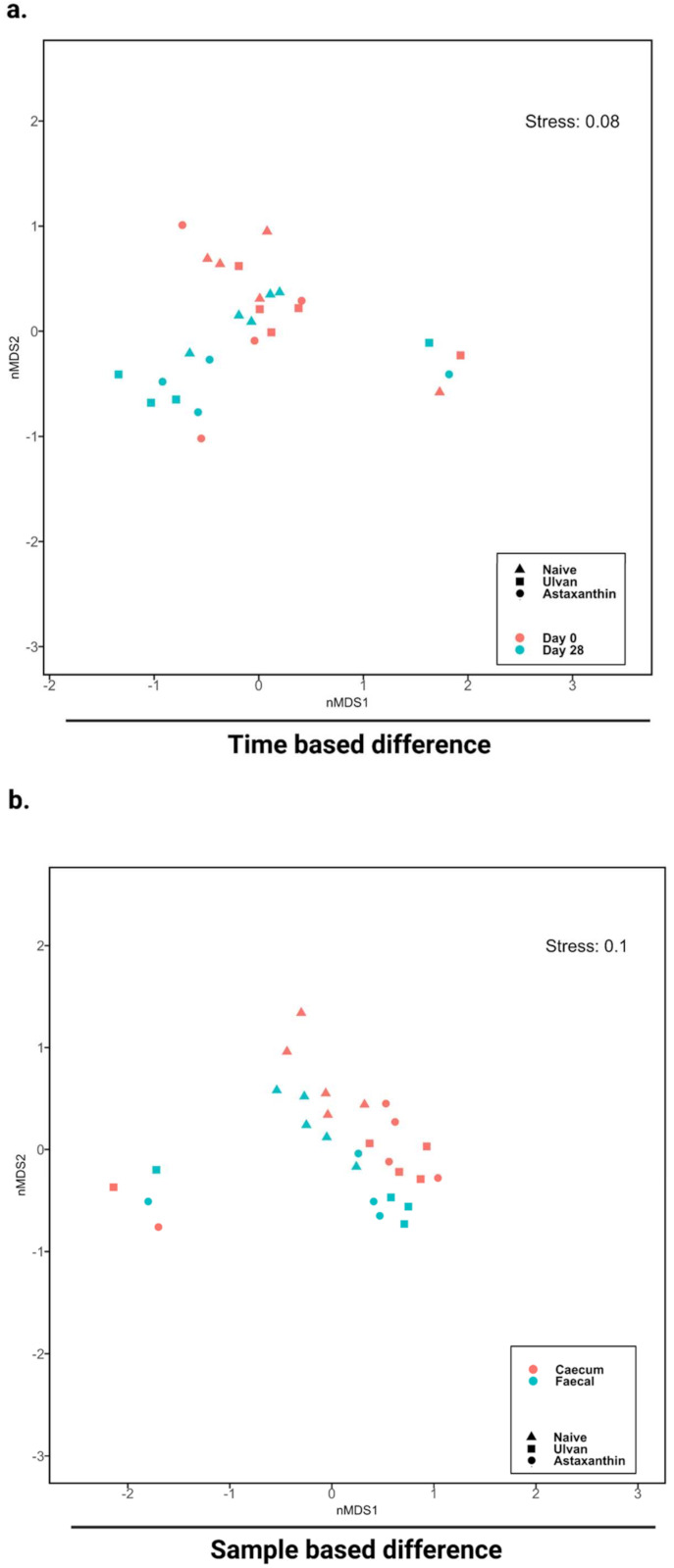
Multidimensional scaling (MDS) plot of bacterial community structure differences upon ulvan and astaxanthin feeding, based on sample type, that is, time-based (**a**) and fecal and caecum samples (**b**), at Day 0 and Day 28.

An effect of ‘time’ was observed on the overall bacterial community structure from fecal samples collected on days 0 and 28 (Figure 3a, Table 1; PERMANOVA: *p* = 0.0422). In addition, an effect of ‘treatment’ was observed on the overall bacterial community structure based on Bray–Curtis dissimilarity irrespective of sample type (i.e., fecal or caecum) on day 28 (Figure 3b, Table 2; PERMANOVA: *p* = 0.0121).

There was statistical support for differences between naïve samples and samples supplemented with astaxanthin or ulvan (Table 3; PERMANOVA: *p =* 0.0039, *p =* 0.0037; respectively), indicating an effect of algal extract feeding on the bacterial community structure.

### 3.4. Taxonomic Structure of the Bacterial Communities after Ulvan and Astaxanthin Feeding

**Figure 4:**
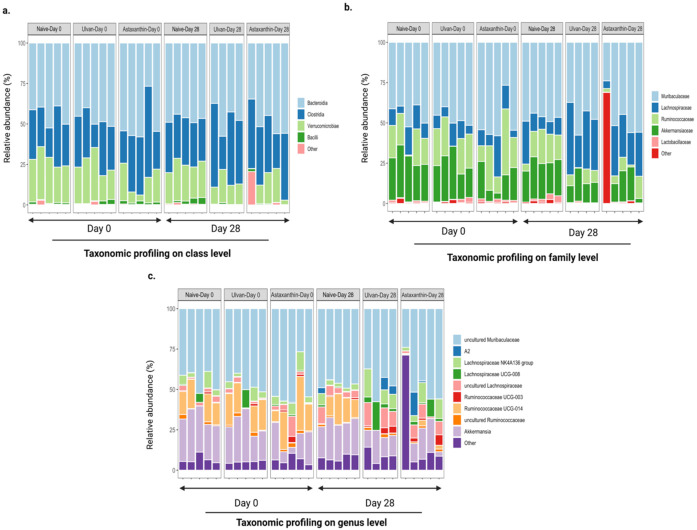
Taxonomic profiles of bacterial communities at class (**a**), family (**b**), and genus (**c**) level of all fecal samples collected from ulvan and astaxanthin-fed mice from Day 0 and Day 28.

The most abundant bacterial classes found in the fecal samples for different treatment groups on days 0 and 28 belonged to the classes *Bacteroidia*, *Bacilli*, *Clostridia,* and *Verrucomicrobia* (Figure 4a). Other bacterial classes were present at lower relative sequence abundance levels in some (but not all) fecal samples both on days 0 and 28 (Figure 4). A higher relative abundance of bacteria from the class *Verrucomicrobiae* was observed in naïve samples (day 0: 26.45% ± 4.40%; day 28: 21.87% ± 2.86%) compared to astaxanthin-fed samples (day 0: 13.97% ± 8.44%, *p* = 0.0331; day 28: 10.93% ± 9.38%, *p* = 0.0293), while a lower relative abundance of bacteria from the class *Clostridia* was observed in the naïve samples (28.28% ± 1.91%) compared to ulvan-fed samples (39.20% ± 13.48%, *p* = 0.0425) on day 28 (Figure 4a).

The bacterial families *Muribaculaceae* (class *Bacteriodia*), *Lachnospiraceae* (class *Clostridia*), *Lactobacillaeceae* (class *Bacilli*), *Ruminococcaceae* (class *Clostridia*), and *Akkermansiacaeae* (class *Verrucomicrobia*) were most abundant in the fecal samples for different treatment groups on days 0 and 28 (Figure 4b). A lower relative abundance of bacteria from the family *Akkermansiaceae* was observed in astaxanthin-fed samples (day 0: 13.97% ± 8.44%, *p* = 0.0331; day 28: 10.93% ± 9.38%, *p* = 0.0293) compared to naïve samples (day 0: 26.45% ± 4.40%; day 28: 21.87% ± 2.86%), while higher relative abundances of bacteria from the family *Lachnospiraceae* was observed in the ulvan-fed samples (33.20% ± 10.31%, *p* = 0.0002) on day 28 compared to naïve samples (12.87% ± 7.86%) (Figure 4a). A higher relative abundance of bacteria from the family *Lachnospiraceae* was observed in the ulvan-fed samples on day 0 (9.07% ± 3.63%) compared to day 28 (33.20% ± 10.31%, *p* < 0.0001). Additionally, on day 28 an increase in the relative abundance of *Lachnospiraceae* was also observed in astaxanthin-fed (12.31% ± 8.30% to 26.29% ± 10.42%) samples. In comparison, the lower relative abundance of bacteria from the family *Ruminococcaceae* was observed in the astaxanthin-fed samples on day 0 (21.42% ± 13.16%) compared to day 28 (6.10% ± 5.11%, *p* = 0.0146) (Figure 4b).

Fecal samples for different treatment groups on days 0 and 28 were abundant in the genus *Akkermansia* and the uncharacterized genera *Muribaculaceae A2*, *Lachnospiraceae NK4A136 group*, *Lachnospiraceae UCG-008*, *uncultured Lachnospiraceae*, *Ruminococcaceae UCG-003*, *Ruminococcaceae UCG-014,* and *uncultured Ruminococcaceae*. A higher relative abundance of bacteria from the genus *Akkermansia* was observed in the astaxanthin-fed samples (13.97% ± 8.44%) compared to naïve and ulvan-fed samples (naïve: 26.45% ± 4.40%, *p* = 0.001; ulvan: 23.53% ± 7.11%, *p* = 0.0155) on day 0, as well as a decrease in the astaxanthin-fed samples (10.93% ± 9.38%, *p* = 0.0034) compared to the naïve samples (21.87% ± 2.86%) on day 28 (Figure 4c). A higher relative abundance of bacteria from the genus *Ruminococcaceae UCG-014* was observed in the naïve samples (11.52% ± 7.13%) compared to the astaxanthin (1.61% ± 1.25%, *p* = 0.0064) and ulvan-fed samples (0.15% ± 0.1%, *p* = 0.0042) on day 28 (Figure 4c). Additionally, a higher relative abundance of bacteria from the genus *Ruminococcaceae UCG-014* was observed in the astaxanthin and ulvan-fed samples on day 0 (astaxanthin: 16.07% ± 13.12; ulvan: 14.69% ± 8.30) compared to day 28 (astaxanthin: 1.61% ± 1.25%, *p* = 0.0002; ulvan: 0.15% ± 0.1%, *p* = 0.0008) (Figure 4c).

## 4. Discussion

Ulvan and astaxanthin supplementation in mice changes the structure of gut microflora compared with naïve control mice, dominated by bacterial populations in the fecal samples belonging to classes *Bacteroidia*, *Bacilli*, *Clostridia*, and *Verrucomicrobia*, and their role has been attributed as a probiotic class of bacteria that can help in maintaining the intestinal barrier in mice and rats. Most of these microbial classes of bacteria have been previously reported for fermenting the polysaccharide into short-chain fatty acids (SCFA’s) and other metabolites in the gut [1,18]. Natural polysaccharides and carotenoids originating from algae have been widely studied for their roles in immune protection [36,37]. However, the effects of algal polysaccharides and carotenoids on gut microbiota regulation have not been extensively studied.

Our study detected an increase in gut microbiota richness with time in our polysaccharide-fed samples compared to naïve samples. Metabolites produced after the breakdown of such polysaccharides may be a source of nutrients for other beneficial bacteria, thus maintaining the gut homeostasis [4]. Polysaccharides are considered important regulators of microecology in the gut, directly affecting the selective colonization of intestinal flora [1]. Furthermore, the family *Lachnospiraceae* (*Clostridia*) was observed in fecal samples, which possess some beneficial xylan/fiber-degrading bacteria, such as *Eubacterium halli*, that have been reported for their butyrate-producing properties and facilitating the degradation of indigestible dietary fiber [38]. Previous studies on mice have shown that the loss of bacteria from the *Lachnospiraceae* family is linked with increased incidences of inflammatory bowel diseases and chronic gastrointestinal tract infections [39]. Additionally, a reduced abundance of *Lachnospiraceae* in an *in vitro* culture of patients with ulcerative colitis was associated with the relapse of disease condition due to low butyrogenesis, leading to ulcerative colitis recurrence [40].

In our study, we observed a reduction in the family *Ruminococcaceae* (*Clostridia*) in ulvan-fed mice with time, which is associated with a healthy gut and previously shown to be upregulated in mice after treatment with polysaccharides extracted from seaweed *Porphyra haitanensis* (Rhodophyta) and *Ulva prolifera* (Chlorophyta) [41]. The reduction of the *Ruminococcaceae* family in our study could be an indicator of the inaccessibility of carbohydrate-binding modules provided by purified extract of ulvan to the gut bacteria [1,41]. Some gut bacterial species are more specific than others regarding substrate specificity and degrade different amounts of glycans based on the available substrate types [1].

The increase in the relative abundance of bacterial populations at the family level in the ulvan and astaxanthin fed groups corroborates the previous reports that dietary feeding of algal polysaccharides or carotenoids could also carry therapeutic value as prebiotic supplements [22,42,43,44]. Various diet regimens have been shown to decrease the impact of the opportunistic bacterial population, that is, by increasing the population of beneficial bacteria and suppressing inflammatory responses in the gut [45]. In accordance, polysaccharides from different origins have been reported for several bioactive properties, including the modulation of the bacterial population in the gut [1]. Polysaccharides isolated from *Pleurotus eryngii*, an edible mushroom species, have been reported to increase the families of commensal bacterial populations, namely members of the families *Lactobacillaceae, Porphyromonadaceae*, *Bacteroidaceae*, and *Rikenellaceae* [46]. In another study, Tang *et al.* reported that diluted and concentrated alkali-soluble polysaccharides from purple sweet potato [*Ipomoea batatas* (L.) Lam] increased the population of *Bacteroidetes*, *Lachnospiraceae*, *Ruminococcaceae*, and *Oscillospira* that produce SCFAs, such as butyric acid, acetic acid, and propionic acid in the mouse gut [43]. Additionally, similar studies have reported that digestion of polysaccharides in the gut can regulate the gut bacterial population and modulate the gut metabolite production to benefit overall gut health [42,47,48].

Additionally, the role of the gut bacterial population in the fermentation of the natural compounds also plays a significant role in maintaining gut homeostasis through SCFA’s and other metabolite production [1]. Bobin–Dubigeon *et al.* reported lower degradation rates and fermentation of ulvan compared to individual sugars in an *in vitro* experiment using human fecal microbiota. However, the sugar constituents (rhamnose, ulvanobiouronate, and glucuronate) were found to be highly fermentable, suggesting that sugars are readily taken up after digestion by the gut microbes [49,50]. Sugar constituents in ulvan have been reported elsewhere for their efficacy in modulating immune responses [8]. Interestingly, a similar study on ulvan fermentation using the human fecal microbiota reported an increased abundance of *Bacteroides*, *Lactobacillus,* and *Bifidobacterium* after 12 h of culturing [34]. Although ulvan from various other Ulva species have been reported for their efficacy in modulating the immune response [5,44,51], our study is the first to elucidate the effect of ulvan from *U. ohnoi* on mouse gut microbiota.

Our study supports the beneficial effect of astaxanthin as demonstrated by an increase in *Lachnospiraceae* families, whose members can ferment dietary substrates to beneficial SCFAs such as butyrate [40]. Based on an increased relative abundance of *Lachnospiraceae*, our findings suggest that astaxanthin can be investigated further for its potential as an immunomodulator and could improve gut health. In a recent clinical study of concordant and discordant cohort of identical twins, the non-allergic cohort were reported to have a higher abundance of the bacterial class *Clostridia,* especially *Lachnospiraceae* or *Ruminococcaceae* in their fecal samples as compared with the allergic cohort of twins [52]. The authors suggest these results indicate a link between the lack of *Lachnospiraceae* or *Ruminococcaceae* and increased allergic sensitization in the group [52]. Furthermore, in previous studies, members of the class *Clostridia* have also been reported to protect peanut sensitized mice [53]. Additionally, ulvan and astaxanthin have been shown in our study to increase the relative abundance of *Firmicutes* that belongs to class *Clostridia*; this provides us a proof of concept to study that these polysaccharides may have beneficial effects as prebiotics.

In immunological aspects, astaxanthin is a potent antioxidant and anti-inflammatory compound that has been widely studied and is used commercially as a nutraceutical [16]. Astaxanthin has a unique structure having both hydroxyl and keto groups attached, providing lipophilic and hydrophilic properties. These properties allow the compound access through the cell membrane, and it can also cross the blood-brain barrier and exert potential effects [16,54]. Astaxanthin, a natural added supplement in food, has been demonstrated to be beneficial *in vitro* and *in vivo* systems against various diseases, such as cancer, obesity, and diabetes [54]. Astaxanthin promotes M2 polarization and macrophage activation in case of inflammation and has been reported for potent anti-inflammatory properties such as inhibiting pro-inflammatory cytokines via NOD signaling pathways in the case of atopic dermatitis [55,56,57,58]. The innate lymphocyte cells (ILCs) are immune cells produced in the intestinal barrier system [59]. Astaxanthin may also assist in ILCs differentiation upon digestion, especially ILC1, which are lymphocytes very similar to Th1 cells and can express pro-inflammatory cytokines, such as TNF-α and IFN-γ upon foreign pathogen interactions in the gut [60]. An *in vitro* study also indicated the role of astaxanthin as a potential anti-allergic compound possessing anti-histamines-like activity to inhibit pathological immune activation of T-lymphocytes in case of allergic rhinitis and seasonal allergies [61].

Astaxanthin has recently gained attention for its role in maintaining immune homeostasis through gut health [22,62]. A recent study in C57BL/6 J mice showed the potential impact of astaxanthin on the cecal gut microbial diversity [22]. This study suggested that the administration of astaxanthin alters the microbial signatures and regulates metabolic homeostasis in a gender-specific manner [22]. Its role in sugar metabolism in a high-fat diet mouse model by boosting the carbohydrate metabolism, lowering the blood glucose level and insulin resistance through maintaining intestinal integrity provided a clue for the potential use of this natural polysaccharide in metabolic disorders [63].

In summary, natural algal extracts such as ulvan and astaxanthin assist the propagation of beneficial microbial populations such as *Bacteroidia*, *Bacilli*, *Clostridia*, and *Verrucomicrobia* in the gut. Furthermore, ulvan and astaxanthin, as described in this study, can improve the relative abundance of the commensal bacterial population in the mouse gut and hence can be further explored as potential prebiotic supplements in future studies.

## 5. Conclusions

Researchers are currently seeking scientific evidence to establish alternative approaches to improve gut health in health and nutrition science. Our study fills this gap by providing an exploratory insight into the microbiome modulation by administration of ulvan from the green seaweed *U. ohnoi* and astaxanthin from the freshwater microalgae *H. pluvialis* in mice. Our results suggest that polysaccharides and carotenoids may contribute to gut health by shaping the gut flora towards the commensal bacterial population. Both ulvan and astaxanthin might exert their effects as a prebiotic in maintaining intestinal homeostasis by regulating the structure and composition of gut microbes. Further research is required to explore the potential of these algal compounds as an alternative therapeutic approach for various metabolic, allergic, and immunological diseases.

## Figures and Tables

**Table 1:** PERMANOVAs based on Bray–Curtis (BC) similarity measure for square-root transformed abundances of all mice fecal samples collected on day 28. *p*-values were calculated using 9999 permutations under a residual model. Bold and * indicates statistically significant values (at alpha = 0.05). df: degrees of freedom; SS: sum of squares; MS: Mean of squares.

Source	df	SS	MS	Pseudo-F	p (perm)	Unique Perms
Treatment	2	1476.6	738.28	1.1211	0.347	9918
Time	**1**	**1599**	**1599**	**2.4283**	0.0422 *	**9938**
Treatment X Time	2	1203.6	601.81	0.91389	0.4955	9932
Res	21	13,829	658.52			
Total	26	18,002				

**Table 2:** PERMANOVAs based on Bray–Curtis (BC) similarity measure for square-root transformed abundances of all mice fecal and caecum samples collected on day 28. *p*-values were calculated using 9999 permutations under a residual model. Bold and * indicates statistically significant values (at alpha = 0.05). df: degrees of freedom; SS: sum of squares; MS: Mean of squares.

Source	df	SS	MS	Pseudo-F	p (perm)	Unique Perms
Sample	1	1218.3	1218.3	1.8142	0.1204	9945
Treatment	**2**	**3657**	**1828.5**	**2.7229**	0.0121 *	**9937**
Sample X Treatment	2	307.19	153.6	0.22873	0.9993	9927
Res	22	14,773	671.52			
Total	27	19,938				

**Table 3:** Pairwise comparison tests between groups. Bold values marked as * are statistically significant based on *p* > 0.05.

Groups	t	p (perm)	Unique Perms
Astaxanthin, Naïve	**1.9029**	0.0039 *	**9939**
Astaxanthin, Ulvan	0.75135	0.6475	9944
Naïve, Ulvan	**2.1882**	0.0037 *	**9936**

## Data Availability

The data presented in this study are available on request from the corresponding author.

## Supplementary Materials

The following supporting information can be downloaded at: https://www.mdpi.com/article/10.3390/foods11040565/s1, Figure S1: Total operational taxonomic unit (OTU) count before and after rarefaction analysis. Rarefaction curves (a) before and (b) after normalization (22,900 sequences).
