# Supplementary material for: The Algal Polysaccharide Ulvan and Carotenoid Astaxanthin Both Positively Modulate Gut Microbiota in Mice
Source: Foods. 2022 Feb 16;11(4):565. doi: 10.3390/foods11040565 (PMC8871025; doi:10.3390/foods11040565)

Figure S1. Total operational taxonomic unit (OTU) count before and after rarefaction analysis. Rarefaction curves (a) before and (b) after normalization (22,900 sequences).

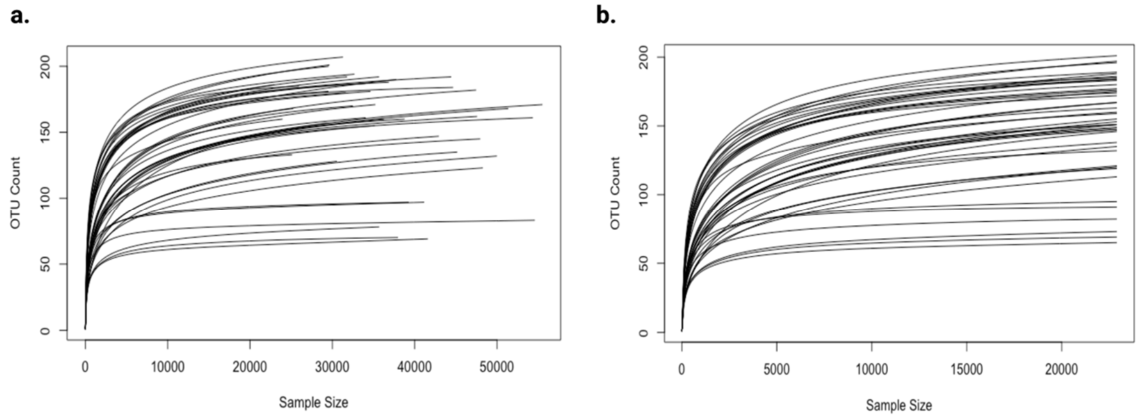

Supplement: Supplementary file 1 [file foods-11-00565-s001.zip › foods-1556854-supplementary.pdf]
